# Pilocarpine in the Pruritus of Pregnancy

**Published:** 1879-09

**Authors:** 


Pilocarpine in the Pruritus of Pregnancy.—"A country doctor" writes to the *British Medical Journal* that a single dose of one-third of a grain of nitrate of pilocarpine, by the mouth, served to bring on profuse sweating and salivation, with complete relief of intolerable and persistent itching, which had lasted throughout pregnacy and recurred after delivery.—*Phila. Med. Times.*

